# Wintertime overlaps between female Antarctic fur seals (*Arctocephalus gazella*) and the krill fishery at South Georgia, South Atlantic

**DOI:** 10.1371/journal.pone.0248071

**Published:** 2021-03-04

**Authors:** Connor C. G. Bamford, Victoria Warwick-Evans, Iain J. Staniland, Jennifer A. Jackson, Philip N. Trathan

**Affiliations:** 1 British Antarctic Survey, High Cross, Cambridge, United Kingdom; 2 University of Southampton, Southampton, United Kingdom; 3 International Whaling Commission, The Red House, Impington, Cambridge, United Kingdom; CSIRO Townsville Australian Tropical Sciences and Innovation Precinct, AUSTRALIA

## Abstract

The diet of Antarctic fur seals (*Arctocephalus gazella*) at South Georgia is dominated by Antarctic krill (*Euphausia superba*). During the breeding season, foraging trips by lactating female fur seals are constrained by their need to return to land to provision their pups. Post-breeding, seals disperse in order to feed and recover condition; estimates indicate *c*.70% of females remain near to South Georgia, whilst others head west towards the Patagonian Shelf or south to the ice-edge. The krill fishery at South Georgia operates only during the winter, providing the potential for fur seal: fishery interaction during these months. Here we use available winter (May to September) tracking data from Platform Terminal Transmitter (PTT) tags deployed on female fur seals at Bird Island, South Georgia. We develop habitat models describing their distribution during the winters of 1999 and 2003 with the aim of visualising and quantifying the degree of spatial overlap between female fur seals and krill harvesting in South Georgia waters. We show that spatial distribution of fur seals around South Georgia is extensive, and that the krill fishery overlaps with small, highly localised areas of available fur seal habitat. From these findings we discuss the implications for management, and future work.

## Introduction

Antarctic fur seals *(Arctocephalus gazella)* were heavily exploited by commercial sealing in the South Atlantic following the initial discovery of their abundance in the Southern Ocean [1]. Populations inhabiting the Sub-Antarctic archipelago of South Georgia (54°17’S 36°30’W) were commercially targeted from 1786 onwards, with the population close to extinction by 1820, and likely extirpated by the end of the 19th Century [2] when the industry shifted its focus to populations further south [1]. However, since the cessation of commercial sealing [3] the population of fur seals breeding at South Georgia has grown rapidly. This archipelago now supports in excess of 95% of the global population [4], and Antarctic fur seals are now regarded as one of the most numerous otariid species in the Southern Ocean [5]. Early population recovery rates were estimated to be as high as 16.8% per year, between the late 1950’s and early 1970’s at South Georgia [6], with rates falling to 11.5% by the late 1970’s [7], and then to 9.8% per year between the late 1970’s and 1990’s [4] as the population presumably approached carrying capacity in this part of its breeding range.

South Georgia is the global epicentre of fur seal breeding [4]; the surrounding productive polar and sub-polar waters of the Scotia Sea support over 50% of the Antarctic krill (*Euphausia superba)* biomass in the Southern Ocean [8], and provide fur seals with access to their primary dietary staple [4, 9]. Conservative consumption estimates suggest that fur seals breeding on South Georgia consume *c*.3.84×10^6^ tonnes of krill each year, with approximately 52% of this taken by males and 48% by females [10]. Interannual breeding success of fur seals is closely linked with the abundance of krill, with low prey availability corresponding with low pup survival rates and production at South Georgia [11, 12].

**Fig 1:**
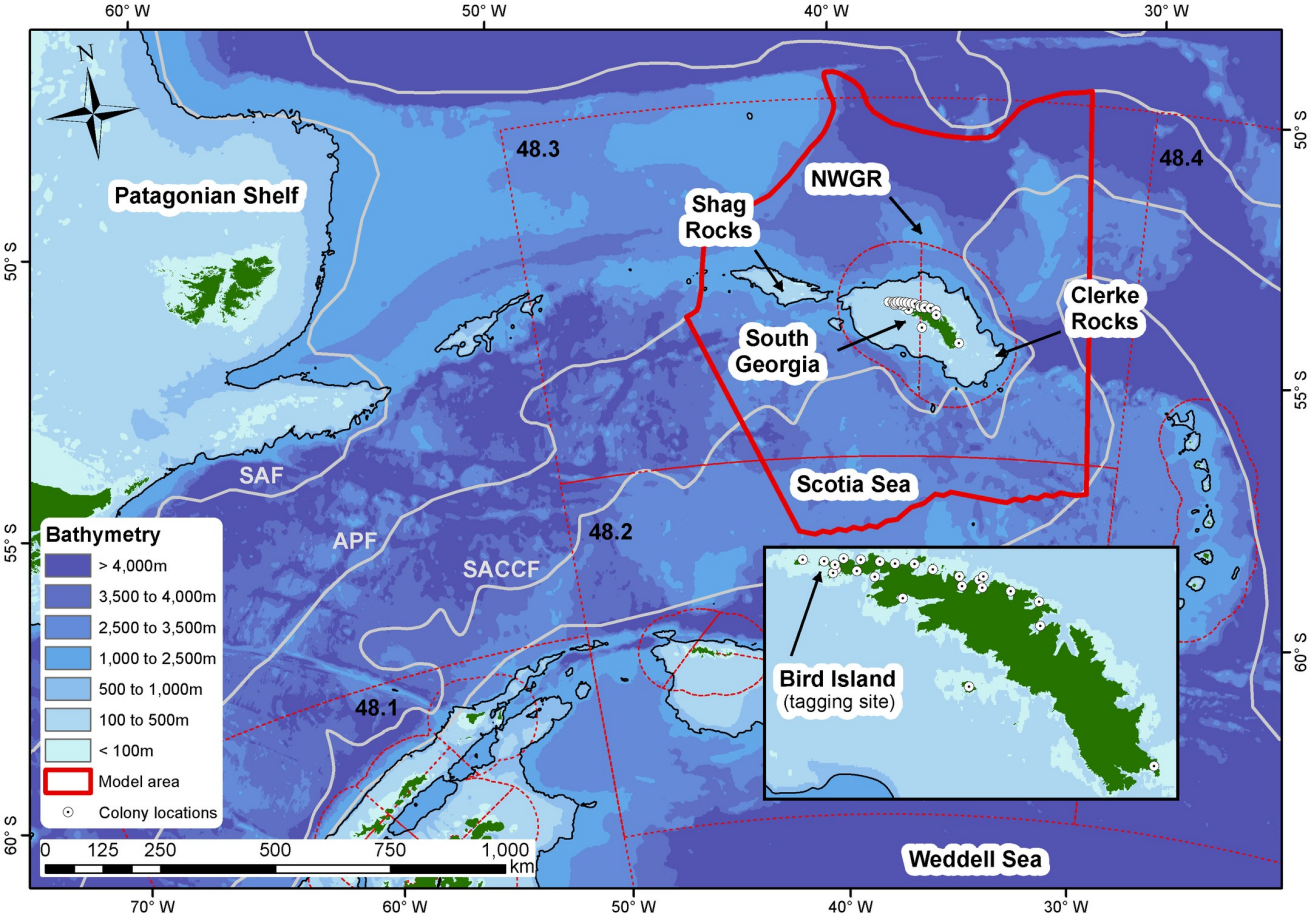
The Scotia Sea region depicting FAO statistical boundaries for CCAMLR Subareas 48.1, 48.2, 48.3, and 48.4. Model area is highlighted in solid red. Major fronts in the Antarctic Circumpolar Current are depicted in grey, and from north to south are: the Sub-Antarctic Front (SAF); the Antarctic Polar Front (APF); the Southern Antarctic Circumpolar Current Front (SACCF); and the Southern Boundary of the Antarctic Circumpolar Current (SBACC). Solid black line denotes the 1000 m isobath, and is indicative of the ‘shelf-break’. Northwest Georgia Rise (NWGR) is indicated. Bird Island (the tagging site) is indicated in the magnified panel. Bathymetry data General Bathymetric Chart of the Oceans (GEBCO).

Recent changes in regional climate dynamics have been linked to recruitment, abundance and distributional changes of krill [13]. Changing environmental conditions are also thought to be related to the number of breeding fur seals at Bird Island, located at the western tip of mainland South Georgia (54°00’S 38°03’W, Fig 1). During the breeding season, lactating female fur seals act as central place foragers, and their ranges are restricted by their need to return to shore to provision their pups [14]. Consequently fur seals, particularly females, are more vulnerable to prey fluctuations during this period [15]. At Bird Island, contemporary counts show a 30% decrease in the number of females between 2003 and 2012 [4, 16]. Since the early 1980s, there has been an increase in the frequency of positive Southern Annular Mode events, which is reflected by warmer sea surface temperatures and reduced krill availability [17]. The phenotypic plasticity of fur seal populations at South Georgia also appear to have shifted in relation to changing climate dynamics [17]. This, coupled with increasing interannual variation in krill supply, may have adverse impacts on breeding success into the future. However, it is also worth noting that population trajectories are now monitored at a second breeding site on South Georgia (i.e. Maiviken; 54° 15’S 36° 30’W), and these may help determine whether observations from Bird Island are localised or representative of an island-wide signal [18]. At present though, the duration of the Maiviken time series is too short for observed trends to be statistically significant [18].

The Government of South Georgia and the South Sandwich Islands (GSGSSI), a UK Overseas Territory, aims to ensure that the harvesting of krill within the South Georgia and the South Sandwich Island Marine Protected Area does not pose a threat to fur seals or other krill predators [19]. Recognising the risks of negative interactions with seals, other krill-dependent predators, and the krill fishery particularly during the summer months [19], the GSGSSI has imposed a series of protective management measures within the South Georgia and South Sandwich Island Marine Protected Area. These include: (i) a closed krill fishing season from October to April, so that the krill fishery is restricted to the winter period when most krill predators are no longer breeding or have seasonally migrated away from the region, and (ii) coastal protection zones to reduce competition with krill predators, based on the foraging range of land-based predators– 22.2 km at Shag Rocks (to the west of mainland South Georgia), 22.2 km at Clerke Rocks (to the east of South Georgia), and 30.0 km around South Georgia itself. More broadly, GSGSSI also implements all management measures agreed by the Commission for the Conservation of Antarctic Marine Living Resources (CCAMLR), the international authority responsible for sustainably managing the Southern Ocean krill fishery and minimising ecosystem impacts.

To facilitate fishery management, the CAMLR Convention Area is divided into Management Areas (https://www.ccamlr.org/en/organisation/convention-area), with each of these further divided into Subareas. The focus of this study is the waters surrounding the island of South Georgia in the South Atlantic (Fig 1), which falls within Subarea 48.3. CCAMLR conducts ecosystem-based management using eight ecological indicator species, which includes fur seals. The use of monitoring species is intended to help identify ecosystem-wide changes. The objective of CCAMLR is to ensure harvesting does not alter ecological relationships, ensuring that any changes in the marine ecosystem as a result of fishing are minimised, and potentially reversible over two to three decades. CCAMLR’s krill management framework is currently in the process of being re-designed (SC-CAMLR-2019), and in the interim a precautionary catch limit of 279,000 tonnes per year for CCAMLR Subarea 48.3 has been set.

Prey consumption by the fur seal population at South Georgia varies seasonally [10], reaching a peak in the winter and a minimum in the summer, when many animals are ashore, fasting for significant periods [10]. Seals require prey to be accessible (i.e. within their dive/depth tolerance), at a certain density and of a given quality [20] to forage successfully. Consequently, factors that affect prey density are likely to impact foraging success, and ultimately reproductive output. Changes in prey density may therefore have important population-level consequences, though females may be more resilient because of their lower *per capit*a consumption [14]. Given the possibility of increased fishing activity in the Scotia Sea [21], and the predicted shift in krill dynamics [13], the extent of any anthropogenic impact on fur seal populations should be evaluated.

Post breeding (*c*.mid-March/April), female fur seals are no longer constrained to return to land [22], and they disperse throughout the Scotia Sea and beyond. During this period there is the potential for interactions with the krill fishery [14, 23]. At South Georgia, this fishery [24] employs two main trawling techniques; traditional mid-water trawls and, more recently, continuous trawling, whereby the krill-catch is continuously pumped from the net cod-end to the ship whilst fishing is underway [25, 26]. Both techniques pose risks of seal: net interactions when underway; a total of 462 fur seals were caught in commercial krill nets between 2003, when reporting first began, and 2005. The introduction of mandatory seal exclusion devices (SEDs) reduced the mortality rate to negligible levels per annum (a single individual in both 2006 and 2007, respectively). However, seals may still be caught in the wings of the net as it collapses just prior to being hauled on board [27]; mortality as a consequence of the krill fishery could therefore be both direct and indirect. Instances of bycatch by the krill fishery in Subarea 48.3 have been described previously [27], and these highlight the need to understand wintertime areas of vulnerability for fur seals in the effort to further reduce bycatch mortalities, a major concern for both CCAMLR and GSGSSI.

Satellite telemetry and associated analytical techniques facilitate investigation into the at-sea distribution and activities of individuals at broad spatial and temporal scales [28, 29]. However, the winter-period is generally under-studied, particularly in the high latitudes, where environmental conditions frequently preclude or restrict predator research. Moreover, limited tag-battery longevity means that few summer tag deployments extend into the winter. Here, using habitat models, we investigate the scale of the spatial overlap between female fur seals tracked from Bird Island during the winter when the South Georgia krill fishery operates (May to September, inclusively). We compare these predictions to the operational footprint and catch recorded by the krill fleet between 1999 and 2019, inclusively, in order to assess the degree of overlap between fur seals and the krill fishery and provide suggestions for the future trajectory of fur seal research and management at South Georgia.

## Methods

### Deployment procedure

Platform terminal transmitters (PTT, Sirtrack, Havelock North, NZ; Kiwisat 101; 245g, 13 x 6.5 x 1.9 cm) were fitted to 14 lactating female fur seals breeding on Bird Island, South Georgia during the late summer; 8 were deployed in 1999 and 6 in 2003 following procedures described in [30]. Capture and restraint times were minimised, and were always less than 2 and 25 minutes, respectively. No anaesthetics were used. PTTs were attached with epoxy resin to the fur of each animal along the dorsal region midway between the shoulder blades [14] to maximise surface exposure and hence signal uplink strength. All animal handling procedures described here were subject to review and approval by the BAS Animal Welfare and Ethics Review Board. No unique identifiers were issued for these procedures.

## Telemetry processing

Data outside the winter period when the krill fishery operates (May to September) were removed, as were invalid location uplinks (ARGOS Quality Z [31]). To aid with state space model convergence, the remaining data were filtered using the R package ‘argosfilter’ [32] to remove improbable positions characterised by unrealistic turning angles, distances and speeds between locations–turning angle thresholds of <15° and 25° were used for distances >2.5 km and 5 km, respectively, and all locations were subject to a speed filter of 10 m s^-1^. The data considered for modelling here represent non-constrained, post-breeding movement activity by female fur seals. During this period, animals are free to move throughout their range without the need to return to land to provision pups. However, whilst free to roam, animals may periodically return to land for rest, although they often rest at-sea. As such, locations which occurred on land were removed, so that the data processed herein represent only at-sea activity. Processed tracks were split if the time between successive uplinks exceeded 3 days [33], in an effort to prevent unrealistic linear interpolations between successive points over unobserved regions. Data were processed in the R package *crawl* [34, 35], where the generation of a random correlated walks in a state-space environment allowed the intervals between the uplinks to be regularised into a defined time step. Here we selected a 3-hour interval to allow inter-tag variations in transmission frequency to be standardised whilst maintaining the inherent properties of each individual track. All subsequent geospatial analysis was carried out in either ESRI’s ArcMap v10.6 (ESRI 2018) or QGIS v.3.10 (QGIS.org, 2021), with data projected into a custom Lambert Azimuthal Equal Area projection centred on 54.01°S 36.3°W. Output files were plotted and visually checked for projection errors.

### Habitat models—Environmental covariates and model selection

To model the relationship between female fur seals and their environment, we used both static and dynamic environmental covariates. Static covariates were derived from high resolution (30 arc-second) GEBCO bathymetric data [36]; in addition to depth, these included slope, standard deviation of the slope, aspect (angle/direction, as if referring to a compass, in which a surface faces), and distance to reported colony locations [37]. All were calculated using the spatial analyst toolbox in ArcMap v10.6. Dynamic covariates pertaining to wind stress curl (ID: erdlasFnWPr_LonPM180, resolution of 1°x1°) and sea surface temperature (ID: erdPH2sstdmday, resolution 0.0417°x 0.0417°) were extracted using the R package ‘rerddapXtracto’ [38], which links to the ERDDAP data servers at the NOAA/SWFSC Environmental Research Division. Monthly composites of chlorophyll-a data were obtained at a resolution of 0.25°x0.25° from the E.U. Copernicus Marine Service Information: Global Ocean Biogeochemistry Hindcast (available at: https://resources.marine.copernicus.eu/). Chlorophyll data were log transformed prior to analysis.

To model both presence (seal locations, given by the output of the models implemented in the *crawl* package) and absence (unobserved locations) of fur seals, three independent pseudo-absence locations were randomly generated for each observed presence point; only data within the modelled area were considered for analysis. This area was bounded in the north by the position of the Polar Front [39, 40] and in the south by an average of the maximum winter sea ice extent (1981–2010) (available from https://nsidc.org/data). Antarctic krill is not the primary prey resource for seals outside these boundaries, as it does not occur north of the Polar front in significant [41], so these boundaries restricted our analysis to krill-driven habitat use patterns. We extracted environmental covariate data for each location (whether observed or pseudo-absence) for model input.

Three models were built for the winter period (May to September inclusive, reflecting the licenced period for the modern krill fishery): for 1999, 2003 combining both years. Collinearity between covariates was tested using Variance Inflation Factors (VIF), with a threshold of 0.7 [42], in the R package *usdm* [43]; of the pair, or pairs, of collinear variables that exceeded this threshold, the variable with the higher of the two scores was removed. In all instances this step highlighted the collinearity between bathymetric slope and its standard deviation; as such the standard deviation was removed in all candidate models. The probability of occurrence of fur seals tracked from Bird Island was modelled using Generalised Additive Models (GAMs) with a binomial error structure in the R package *mgcv* [44]. Smooths of each covariate were taken by fitting cubic regression splines with shrinkage, whilst knots were set to 4 to minimise overfitting [44].

*K*-fold cross validation was used to evaluate model performance, where *K* equalled the number of months in each year’s winter period (data were available May through August in 1999, and May through September in 2003). For each individual period, models were built using data from one month and evaluated using data from the remaining months of that period. Model evaluation applied area under the curve (AUC), sensitivity and specificity (correctly predicted presences and absences, respectively) scores generated in the R package *pROC* [45]. Values range from 0.5 to 1.0, where the lower bound indicates predictive performance no better than random, and 1.0 a perfect model. A forward stepwise approach was applied during model selection. Each covariate was ranked and the highest selected; the remaining covariates were added iteratively until the overall model AUC value did not increase. Semi-variograms produced in the R package *gstat* [46] revealed that spatial autocorrelation was present to some extent in the data (< 2km), and thus the cross-validation approach applied here provides a cautious means of selecting modelled covariates [47], which was unlikely to affected by the spatial autocorrelation. Selected models were then applied to predict the foraging distribution of fur seals from all colonies on the north coast of South Georgia using the location of breeding sites detailed in [37].

### Spatial overlap processing

The predicted likelihood of occurrence for two threshold levels (>95% and >50% likelihood of seal occurrence) were used to define the overlap of the fishery with predicted seal occurrence for each model, after [48, 49]. Fishery data (metric tonnes, t, caught) for Subarea 48.3 for the years 1999 to 2019 were provided by CCAMLR. We accounted for known spatio-temporal location issues associated with records of continuous fishing (i.e. the allocation of a continuous fishing effort to a single Latitude/Longitude position) [50] by using the start position for each haul. Fishery data were collated for the winter period (May to September), and summarised using per unit area (km^2^) in regions that overlapped with the modelled thresholds. We used kernel densities of the krill fishing data to illustrate the spatial overlap between the fishery footprint and two thresholds of model predictions (>95% likelihood and >50% likelihood) using the R packages ‘MASS’ within ‘ggplot2’ [51, 52] (Fig 3).

## Results

### Model selection, performance and evaluation

Telemetry data from 14 individual female fur seals were modelled (mean combined tag duration 76 ± 52 days [1999: mean tag duration 45 ± 28 days; 2003: mean tag duration 116 ± 49 days]; Table 1). For each year and period, AUC values showed that the variables that best described the distribution of female fur seals were distance from the colony (CDIST–Table 2). The model describing the winter of 1999 showed an increase in AUC values after the addition of sea surface temperature and depth as covariates. The model for the winter of 2003 showed that the use of four covariates most accurately described fur seal distribution, whilst the combined year model required five covariates to best describe fur seal distribution. All models included the covariate distance to colony. For model development, distance to colony was based on distance to Bird Island. However, for the spatial extrapolation of these models, this variable was substituted with distance to the colonies reported in [37], to more accurately predict across the entirety of coastline of South Georgia. The AUC, specificity, and sensitivity values for all models suggested that all models performed well (>0.8) at predicting seal presences and absences.

**Table 1:** ARGOS locations for each tagged seal, start date, end date, and durations for fur seals instrumented in 1999 (n = 8) and 2003 (n = 6).

Deployment year	ID	Number of ARGOS locations	Start date	End date	Tag duration (days)
1999	W5727_9377	282	01/05/1999	06/07/1999	66
	W5757_6074	415	01/05/1999	22/06/1999	52
	W5767_9375	181	01/05/1999	18/06/1999	48
	W5773_9374	158	02/05/1999	22/05/1999	20
	W5793_6076	78	01/05/1999	04/08/1999	95
	W5797_6072	95	01/05/1999	19/06/1999	49
	W5801_9373	97	01/05/1999	13/05/1999	12
	W5811_9301	169	01/05/1999	22/05/1999	21
				mean	45
				sd	28
2003	W6478_1527	143	28/05/2003	15/06/2003	18
	W6479_30205	913	01/05/2003	30/09/2003	152
	W6876_30204	99	19/05/2003	26/09/2003	130
	W6879_30206	1023	06/05/2003	12/09/2003	129
	W6880_30203	254	08/05/2003	30/09/2003	145
	W6881_30201	480	01/05/2003	31/08/2003	122
				mean	116
				sd	49
				combined mean	76
				combined sd	52

**Table 2:** Covariates included in the GAMs and model performance metrics; Area Under the Curve (AUC), specificity (correctly predicted absence locations); sensitivity (correctly predicted presence locations).

Model	Covariates	AUC	Specificity	Sensitivity
1999	CDIST	0.860	0.868	0.775
	DEPTH	0.782	0.893	0.665
	CHL	0.764	0.915	0.653
	CURL	0.756	0.861	0.647
	SST	0.678	0.561	0.783
	SLOPE	0.591	0.552	0.644
	ASPECT	0.565	0.607	0.547
	**CDIST + SST + DEPTH**	**0.877**	**0.849**	**0.819**
2003	CDIST	0.764	0.633	0.816
	DEPTH	0.731	0.721	0.724
	CHL	0.706	0.671	0.755
	CURL	0.682	0.740	0.709
	SST	0.627	0.396	0.905
	SLOPE	0.585	0.645	0.525
	ASPECT	0.551	0.636	0.490
	**CDIST + DEPTH + CHL + SST**	**0.822**	**0.719**	**0.825**
All	CDIST	0.773	0.631	0.809
	DEPTH	0.748	0.791	0.654
	CHL	0.708	0.695	0.715
	CURL	0.681	0.616	0.808
	SST	0.586	0.429	0.834
	SLOPE	0.571	0.685	0.467
	ASPECT	0.553	0.714	0.408
	**CDIST + DEPTH + SST + ASPECT + SLOPE**	**0.806**	**0.677**	**0.791**

Covariate abbreviations as follows: Distance from colony (CDIST); chlorophyll concentration (CHL) and sea surface temperature (SST). Bold face indicates the covariates and their AUC, specificity and sensitivity values for the best fitting model for each period. Further details of each stage of cross-validation, and individual variable contributions are available in the S1-S3 Tables in S1 File.

### Model response curves

**Fig 2:**
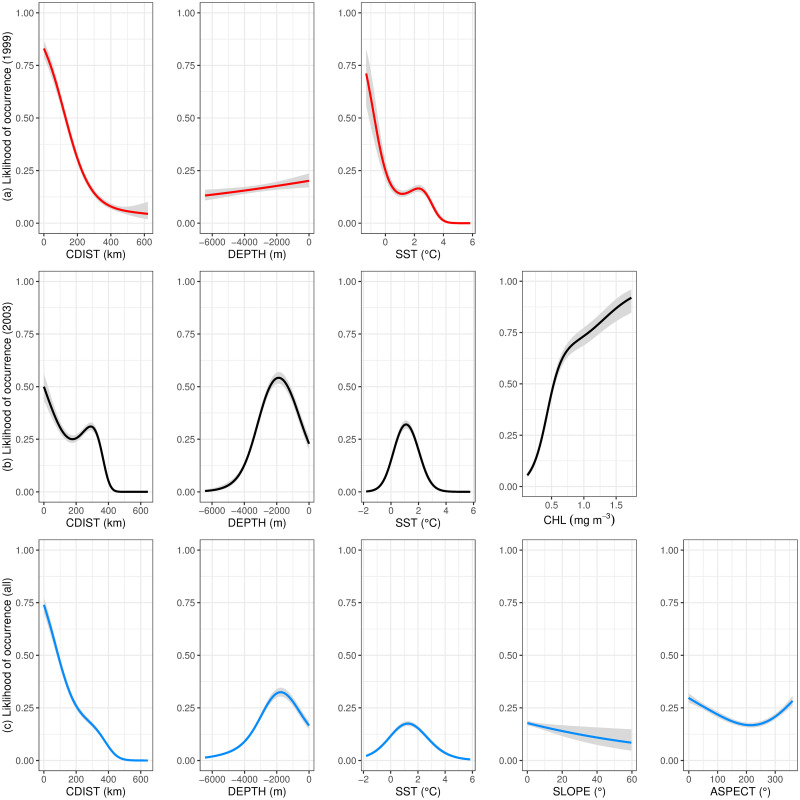
Covariate response curves for the three modelled periods: From top to bottom the plots correspond to the covariates included in the (a) 1999 winter model (red), (b) the 2003 winter model (black), and (c) the all data model (blue). Grey shading indicates ± 2 standard error bounds for models covariates. Plot produced in R package ‘visReg’ [53].

In the 1999 winter model, fur seal distribution was explained by both static and dynamic environmental covariates, with the probability of occurrence reduced as distance from the colony and sea surface temperature increased, a linear decrease in likelihood of occurrence as depth increased (Fig 2a). The 2003 winter model also showed that the probability of occurrence broadly decreased as distance from the colony and depth increased, with probability peaking at *c*.200 km from the colony, and at *c*.2000 m. Dynamic covariates for this period indicate that fur seals preferentially targeted areas where sea surface temperature ranged between 0°C and 2°C, and where chlorophyll concentrations were >0.5 mg m^-3^, (Fig 2b). For the model based on both years, the likelihood of fur seal occurrence peaked at depths c.2000m and between 0°C and 2°C. The results showed a decreasing trend when both distance from the colony and slope increased. These data revealed that fur seals were more likely to occur in regions where the slope is shallower, and where aspect was close to 0° (i.e. more northerly facing) (Fig 2b).

### Spatial predictions and fishery overlap

**Fig 3:**
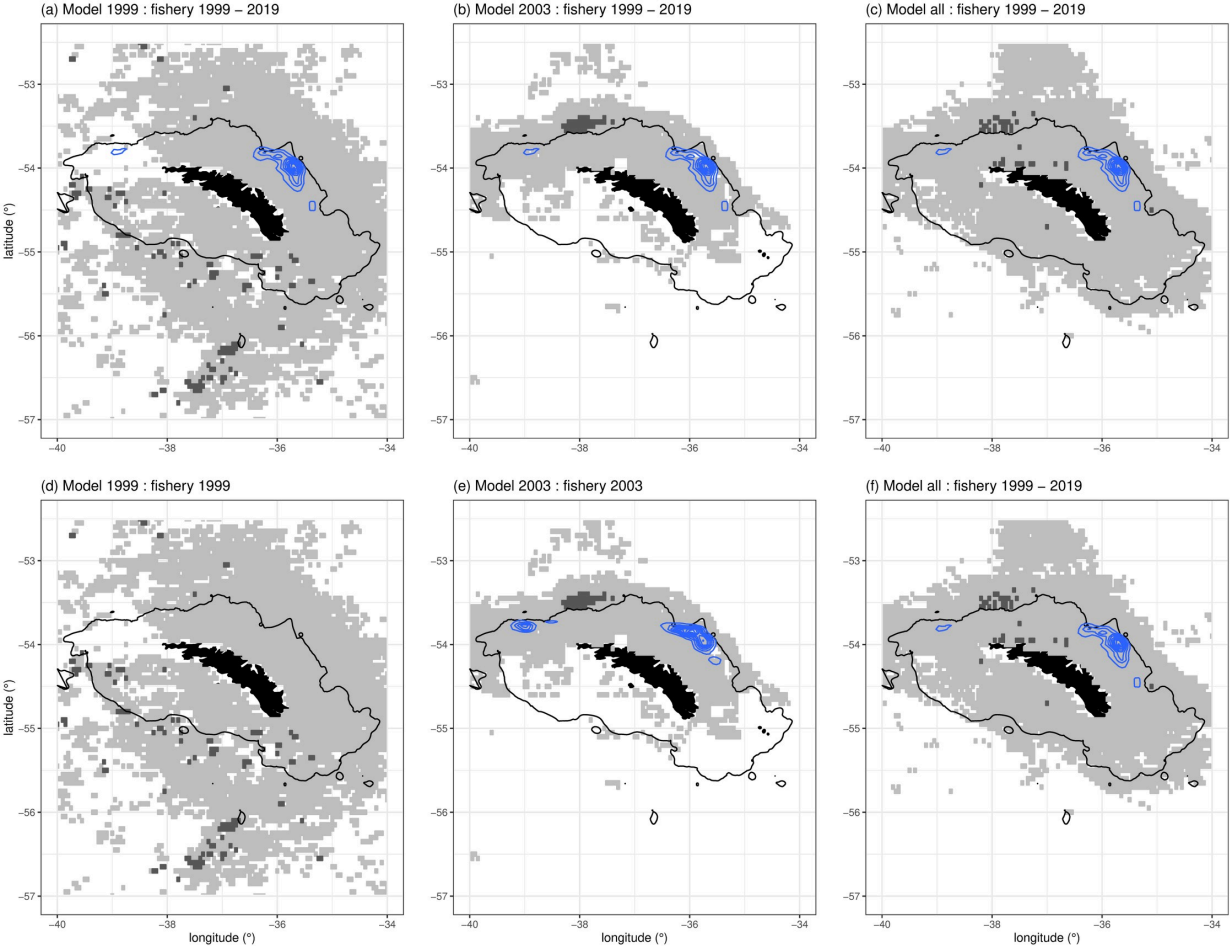
Percentiles of the predicted at-sea distribution of female Antarctic fur seals, where dark grey indicates >95% likelihood of a seal being present, and light grey indicates >50% likelihood. CCAMLR krill catch data summarised by blue kernels. The top row depicts krill catch data from the entire period 1999 to 2019 compared to the modelled fur seal thresholds, whilst the second row depicts total krill catch from the year of the model. Black line corresponds to the 1000m bathymetric contour and is indicative of the South Georgia shelf-break.

**Fig 4:**
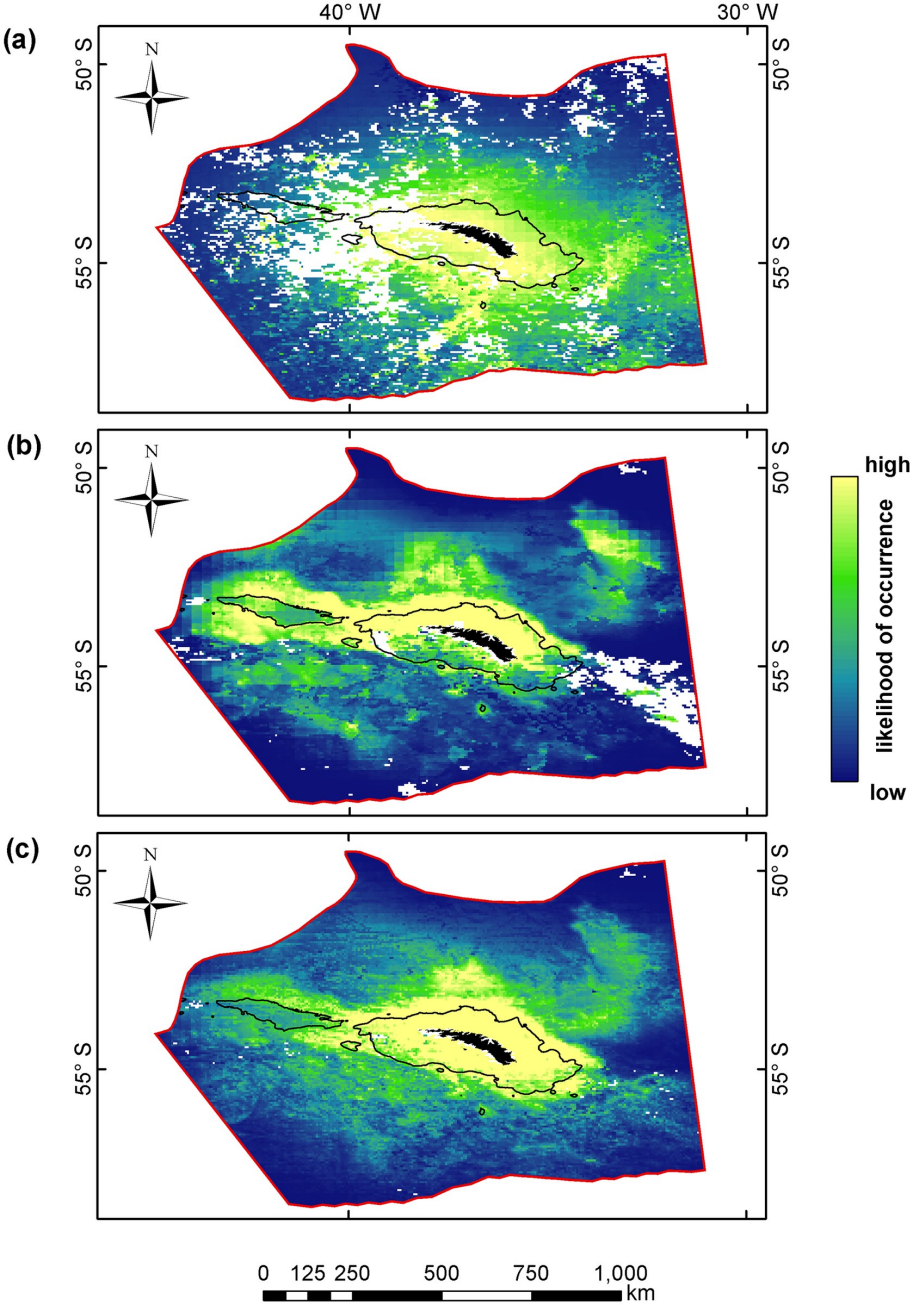
Spatial predictions of the GAM models for (a) winter of 1999 (May to August); (b) winter of 2003 (May to September); and (c) combined winter of both years (May to September). Red line denotes the boundary of the modelled area, bounded by the Polar Front to the north and the average position of the maximum winter ice extent between 1981–2010 to the south. Black line denotes the 1,000m bathymetric contour, which indicates the shelf-break of South Georgia. Blank cells indicate areas where there were gaps in the remotely sensed covariate data, and thus no prediction in these areas.

The spatial footprint shows that over the period 1999 to 2019 the krill fishery primarily operated on the northern shelf break of South Georgia, specifically to the north-east of Cumberland Bay, 54°13 S 36°26 W (Fig 3a–3c). This distribution falls within the predicted distribution of female fur seals in all modelled time periods. However, the footprint of the fishery rarely overlapped with the higher, >95% likelihood, threshold of fur seal occurrence–in all instances the fishery primarily overlapped with the lower, >50% likelihood, threshold. Between 1999 and 2019, the krill fishery caught between 85–97% of their catch within regions modelled to have a >50% likelihood of female fur seals being present (Table 3). In years contemporaneous to the modelled tracking data (Fig 3d–3f), the krill fishery only operated in the vicinity of South Georgia during 2003 (in 1999 effort was further south), resulting in an extraction of between 83–95% of their catch within modelled areas corresponding to a >50% likelihood of a seal being present. The fishery did not overlap with the higher threshold regions in that year. These results show that the spatial distribution of the krill fishery is highly focussed, occupying only a fraction of the modelled area (Fig 3). Fishery activity, per unit area, was focussed on between 8–24% of predicted fur seal distribution (Table 3). Data presented here suggest that vessels operate in areas of known krill abundance, targeting similar areas year-on-year, where they extract the majority of their catch. In all periods considered, the krill fishery took the majority of its catch from Subarea 48.3 within the vicinity of South Georgia, and within regions predicted to have a >50% likelihood of fur seals being present. However, it is clear that fur seal distribution extends into regions far beyond the footprint of the krill fishery (Fig 4). The winter of 1999 was characterised by a dispersal of fur seals into the wider South Georgia region, beyond the shelf-break, in a uniform pattern around the island (Fig 4a). In contrast, in 2003, seals appeared to display a much stronger affinity towards the shelf, and to the north/north-western aspects of South Georgia (Fig 4b). In the combined model (Fig 4c), the spatial distribution of fur seals clearly showed a strong preference for the shelf-region of South Georgia; these areas of preference extend to the west along the 1,000m contour, over the North-West Georgia Rise, and also offshore to the north-east of South Georgia.

**Table 3:** Spatial overlap of the krill fishery and model predictions of the likelihood of occurrence of female fur seals during the winter months (May to September, inclusively) at two threshold levels; >95% likelihood, and >50% likelihood of occurrence.

**Model year**	**Model prediction threshold**	**Model area (km**^**2**^**)**	**(a) Overlap with fishery catch recorded concurrent to tracking data**			
			**Fishery footprint (km**^**2**^**)**	**Total catch (t)**	**Fishery footprint as a proportion of modelled area (%)**	**As a proportion of total annual catch in subarea 48.3 (%)**
1999	50th	148920.18	-	-	-	-
	95th	3092.04	-	-	-	-
2003	50th	55889.86	2,965.52	54,937.10	24.22	83.04
	95th	738.91	-	-	-	-
all	50th	85452.69	3,164.82	62,574.96	23.36	94.58
	95th	735.06	-	-	-	-
**Model year**	**Model prediction threshold**	**Model area (km2)**	**(b) Overlap with all fishery catch recorded between 1999 and 2019**			
			**Fishery footprint (km**^**2**^**)**	**Total catch (t)**	**Fishery footprint as a proportion of modelled area (%)**	**As a proportion of total catch (1999 to 2019) in subarea 48.3 (%)**
1999	50th	148920.18	11,219.15	696,861.86	7.53	84.93
	95th	3092.04	-	-	-	-
2003	50th	55889.86	12,242.64	720,703.05	21.90	87.84
	95th	738.91	73.79	108.40	9.99	0.01
all	50th	85452.69	13,546.50	794,767.52	15.85	96.86
	95th	735.06	91.71	796.22	12.48	0.10

^(a)^ Describes these overlaps for the catches landed in the year contemporaneous to the model predictions.

^(b)^ Describes the overlaps between the modelled thresholds and all catches taken within the predicted area between 1999 to 2019. Dashes indicate that there was no overlap between predicted fur seal distribution and the krill fishery. Note there was no active krill fishery during the winter of 1999. Total krill catch in subarea 48.3 between 1999 and 2019 was 820,511 tonnes, and in 2003 was 66,159 tonnes.

## Discussion

Here we present an analysis of the spatial overlap between female Antarctic fur seals and the winter krill fishery that operates around South Georgia, in CCAMLR Subarea 48.3. Using PTT data from 14 individuals we model, for the first time, the winter distribution of post-breeding female fur seals and discuss implications for management. Over recent years, the krill fishery has operated around the northern shelf-break of South Georgia [18, 24], which when examined alongside the predictions from our models, shows that there is a substantial overlap between likely fur seal distribution and the footprint of the fishery. Understanding spatial interactions between two such potentially competing entities can inform mitigation strategies, helping to develop a more sustainable use of the system into the future. This is crucial in providing baseline understanding about fur seal habitat use in the winter, and is particularly relevant if the pressure from the fishery increases [54, 55].

### Interannual fur seal habitat

Within the modelled area, female fur seals showed close affinity to the shallower waters of the South Georgia shelf and shelf break, and were predominately found within 500 km of the coast throughout the winter period. While some female seals do migrate to other areas, such as to the Patagonian Shelf or to the ice edge [22, 23], here we have on the *c*.70% of the population that remain south of the Polar Front [56] to feed, primarily on krill, during the winter. We observed a variation in the spatial distributions of seals between years. In the winter of 1999, fur seals were associated with the shelf of South Georgia, but also dispersed into offshore regions. In 2003, fur seal distribution was more contracted than in 1999, with regions beyond the shelf-break being less used, and shelf-regions dominating the predictions. The combined model indicated that the aspect of the bathymetric slope played a role in influencing fur seal distribution, which probably reflects the known north-coast preference of fur seals at South Georgia [37], alongside a spatial bias stemming from tag deployment from a single, north-coast site. Areas of higher likelihood of occurrence were broadly consistent throughout the each model, with an association with the Northwest Georgia Rise (NGR, 52.75°S 37.32°W, Fig 1) being clearly present in both the 2003 and combined models (and to a lesser extent in 1999). Affinity to the NGR may be due to productivity; this region is commonly associated with increased chlorophyll concentrations [57, 58], which in part may be due to the formation and retention of cyclonic water masses, known as Taylor columns above this bathymetric feature [59]. Distance to colony was significant in all models, with this association possibly reflecting a carry-over of learned behaviour from the summer, where seals develop knowledge of prey distribution close to their breeding beaches prior to dispersing more widely.

The climate of the Scotia Sea is highly variable [60], and linked to the cycles of both the El-Niño Southern Oscillation (ENSO) and the Southern Annular Mode [61], which strongly influence interannual krill population dynamics [62]. ENSO has been shown to propagate into the South Atlantic along a 4–5 year lag cycle [63], whist Southern Annular Mode events have been linked to localised changes in conditions at South Georgia [11], and with fluctuations in fur seal populations [16]. These climatological processes, whilst not investigated directly here, may well have influenced the observed interannual differences between 1999 and 2003. However, we cannot exclude that such differences may also have arisen from variations in sample size and individual habitat preference. Examination of patterns of individual, or interannual habitat usage, through further longer-term winter-tag deployments, would be a valuable avenue for future research, and would address a significant gap in our understanding of this system during the winter.

### Model performance and caveats

Performance of all models was good, with AUC, sensitivity and specificity values all falling within commonly accepted bounds. Model predictions of the spatial extent of habitat utilisation by fur seals are consistent with observations from previous studies [22, 23, 64–66], although the spatial scales of these works vary. Our models are based on 14 tracked female seals, which inevitably leads to individual variability in the data, a common issue in such pooled-data modelling exercises. Generalised Additive Mixed Models (GAMMs) offer the ability to account for individual variability within their structure [44]. However, such a framework can prove to be prohibitively computationally demanding and can be difficult to apply to small samples [67], notably the case here for tracks that were divided into several subsets due to gaps in transmission. Consequently, this approach was not followed and standard GAMs were applied. Tag deployment from a single location may also have caused a spatial bias in the proportion of winter overlap between the seals and the fishery, with these models built on data reflecting localised conditions at the west end of South Georgia. Therefore, the models may accurately translate to conditions elsewhere around the island. However, it should be noted that the majority of the population is concentrated in the west of the island [37]. When extrapolated, these models may not fully encompass the true extent of fur seal distribution at the eastern end of the island, particularly given the known differences in foraging behaviour between east and west [66]. As a result, and considering also our sample size representing approximately ~0.0009% of the population, the overlaps with the krill fishery presented here are highly conservative, with the true extent likely to be considerably higher.

### Fishery overlap

Visualisation of the CCAMLR catch data clearly illustrate that the operational footprint of the fishery is focussed on the northern shelf, and shelf-break of South Georgia (Fig 3), with the exception of 1999, when no fishing took place [also see 18]. Our models show that the krill fishery occupied a much smaller footprint compared to the total habitat available to female fur seals during the winter months. Overlaps accounted for *c*.7.5% of the predicted space utilised by seals (at a predicted likelihood threshold of 50%) during years when they are more dispersed (Fig 4a–winter 1999), and up to *c*.21.9% when seals are coastally distributed (Fig 4b–winter 2003), when compared to all fishery data 1999–2019. Overlap was higher in years when comparisons were made to concurrent fishery data (Table 3a). The degrees of overlaps were probably influenced by interannual variation in fur seal distribution, with the probable interaction between seals and the fishery being much higher if both the fishery and seals were to occur in only shelf regions.

Between 1999 and 2019, the krill fishery was operationally constrained to shelf regions, whereas seals ranged further offshore. Regions to the north-west, near to the Northwest Georgia Rise, were especially targeted by seals. In contrast, the fishery repeatedly targeted easterly regions where higher densities of krill have been recorded and caught [68]. These targeted regions closely align to areas where seafloor topography and current regimes are thought to drive aggregations of higher krill biomass [60, 69]. The concentration of fishing effort in shelf regions will result in greater overlap between seals and the fishery in years where fur seals themselves are more coastally distributed. This could lead to localised resource depletion, which likely will have negative consequences for both seals and the fishery through both the removal of krill biomass as well as possible disruption of krill swarms, making seal foraging more energetically costly.

Over the two years that data were available, fur seals displayed differing foraging strategies. In 1999 animals were more dispersed, whilst in 2003 animals showed greater affinity for the shelf-break. Both dispersal scenarios highlight that fur seals from South Georgia are able to range widely over much of the South Georgia and Subarea 48.3 region. Therefore, in doing so, seals directly overlap with where the krill fishery primarily operates. The majority of the krill catch is taken once the vessels arrive on the fishing grounds, early in the season, and as a result overlap with seals in coastal waters occurs when the seals are in a state of post-breeding recovery. Further development of krill fishery management in these waters should therefore consider the post-breeding needs of female seals and their need to recover body condition. This should include consideration of movement of krill, and krill flux, into and out of the South Georgia system, as this is not well understood. Current understanding indicates that ocean currents move krill from the Antarctic Peninsula northwards to South Georgia [70], and then along the northern coast, where localised flows circulate water on the shelf and lead to retention [71]. This translates into higher krill density on-shelf, and at the shelf-break, compared to off-shelf regions [60]. Surface currents around South Georgia are complex, and variable through the year [71]. In the summer, they are predominately westerly, potentially moving krill from the eastern end of the island west along the north coast. However, later in the season the intensity of the westerly current diminishes and an easterly current arises at the north-west end of the island [71]. Typically, summer krill biomass decreases towards the western end of the island [60, 72], which means that the highly localised operation of the fishery midway along the north coast could, in poor-krill years, lead to reductions in available krill biomass, potentially impacting krill predators foraging in the western areas in subsequent months. A re-distribution of the effort and intensity of the fishery over a wider area should minimise any such effect. The western end of South Georgia hosts the majority of the fur seal population, which leads to greater predator-induced depletion of krill in this region [72]. However, we suggest that the impact of any alterations to the operation of the fishery, or its spatial intensity, be examined before implementation. This would be particularly important for periods where krill availability was low. The fur seal population at South Georgia consumes significant quantities of krill, having recovered from historical exploitation [10]. At present the population consumes more than an order of magnitude more krill than the fishery harvests, and prevention of bycatch is well managed. To prevent future impacts, which may occur if the fishery expands, further work to ensure continued effective ecosystem-based management should be planned now; this should include research into krill dynamics, distribution and abundance at South Georgia and the development of coherent long-term, multi-site monitoring and tagging programs, year round.

The GSGSSI have recently updated the management of the South Georgia krill fishery, restricting the season to May through September (previously restriction was between October and March) [73]. As pups and post-breeding mothers appear to remain close to their colony in the first few weeks post-weaning (*c*.mid-March/April), before moving further afield as the winter progresses, additional changes in the temporal distribution of fishing should be considered carefully as so not to put undue pressure on these animals. While the reduction in the fishing period is overall anticipated to be positive for post-breeding fur seals, a winter focus of the fishery means that female fur seals are more dispersed at the point when the fishing begins, and therefore are more likely to overlap with areas of fishing activity. This would increase the likelihood of interactions occurring, however, more tagging efforts are required to confirm this.

## Areas for future consideration

Understanding the spatial distributions of predators in relation to possible sources of conflict with human activities allows important regions to be identified and appropriate management and conservation actions to be taken. However, to improve the overall effort in line with both objectives, functional overlaps need to be considered, rather than simply considering spatial overlaps. There is therefore a need to determine whether the operation of the krill fishery alters the efficiency of predator foraging (negatively or positively), and thus determine any effect on population trajectories. In order to achieve this, future efforts need to address several key gaps in current understanding about predator-prey dynamics: (i) An up-to-date fur seal population assessment is required–the most recent published estimate was carried out nearly three decades ago, and reflects a year where breeding success was poor [37], so almost certainly does not reflect the current population; (ii) Further work to develop our understanding of the level of overwinter residency of fur seals at South Georgia is needed. Previous studies have shown that whilst many female seals remain at South Georgia, others range over the wider Scotia Sea and beyond [14, 22, 23, 56]. Developing a greater understanding of the proportion of animals which remain versus those which leave South Georgia through an extended year-round multi-site monitoring and tagging program will help to quantify the possible overlap with the krill fishery and establish the level of predator-induced krill removal; (iii) Quantifying the standing stock, and flux of krill into South Georgia waters throughout both the summer and winter seasons remains key to understanding krill-predator-fishery competition. Predator consumption estimates [10] suggest that a high rate of influx is required to sustain the krill supply above the standing stock of 1.5x10^6^ tonnes [74]; (iv) There is a need to develop an understanding of whether or not predators actively target specific swarm densities of krill, whether age-class influences prey-selection, and how these strategies compare to those targeted by the fishery [68]. In addition, there would be merit in examining the effect of the fishery on krill swarm structure, and to identify whether predators are attracted to or avoid regions with an altered krill structure; (v) Finally, future research should move towards establishing a network of multiple multi-year study sites which would lead to more informed island-wide recommendations capable of detecting interannual and long-term variability into the future.

## Supporting information

S1 File (DOCX)
