# Supplementary material for: Wintertime overlaps between female Antarctic fur seals (Arctocephalus gazella) and the krill fishery at South Georgia, South Atlantic
Source: PLoS One. 2021 Mar 4;16(3):e0248071. doi: 10.1371/journal.pone.0248071 (PMC7932113; doi:10.1371/journal.pone.0248071)
Supplement: S1 File — (DOCX) [file pone.0248071.s001.docx]

**Supplementary information - Wintertime overlaps between female Antarctic fur seals (*Arctocephalus gazella*) and the krill fishery at South Georgia, South Atlantic.**

**Telemetry processing**

Argos Platform Terminal Transmitters (PTT) tracking data from 14 female Antarctic fur seals (8 in 1999 and 6 in 2003) tagged at Bird Island in the summer months were examined, and those tags which remained active and transmitting into the winter period (May to September, inclusively) were selected. In order to examine non-constrained, winter behaviour of female fur seals, pupping data available from the British Antarctic Survey data repository were examined to determine whether the tagged female had raised a pup in the tagged year. Tracking data were selected from 120 days after the first observation that a mother-pup were associated, which equates to the approximate time-to-weening period for this species (Forcada and Staniland 2009). If females were still sighted with a pup after 120 days, then, conservatively, an additional 30 days of tracking data were disregarded, in order for analysis to be based on non-constrained female activity. Data used herein corresponded to those uplinks from May to September, inclusively (where available).

Table S1. Covariate performance metrics used to inform variable selection for the winter of 1999 GAM model: Area Under the Curve (AUC), Specificity (correctly predicted absence locations); Sensitivity (correctly predicted presence locations). Covariate abbreviations as follows: Distance from colony (CDIST); chlorophyll concentration (CHL) and sea surface temperature (SST). Bold face indicates the covariates and their AUC, specificity and sensitivity values for the final model for each stage of cross-validation.

| **1999** | | | |
| --- | --- | --- | --- |
| **Variable** | **AUC** | **Specificity** | **Sensitivity** |
| **CDIST** | **0.8596** | **0.8677** | **0.7752** |
| DEPTH | 0.7818 | 0.8927 | 0.6651 |
| CHL | 0.7640 | 0.9147 | 0.6530 |
| CURL | 0.7561 | 0.8612 | 0.6472 |
| SST | 0.6779 | 0.5610 | 0.7830 |
| SLOPE | 0.5908 | 0.5521 | 0.6438 |
| ASPECT | 0.5651 | 0.6069 | 0.5469 |
| **Variable** | **AUC** | **Specificity** | **Sensitivity** |
| **CDIST+SST** | **0.8758** | **0.8567** | **0.8027** |
| CDIST+DEPTH | 0.8612 | 0.8301 | 0.8096 |
| CDIST+SLOPE | 0.8590 | 0.8658 | 0.7748 |
| CDIST+CHL | 0.8585 | 0.8540 | 0.7774 |
| CDIST+ASPECT | 0.8574 | 0.8569 | 0.7752 |
| CDIST+CURL | 0.7896 | 0.8306 | 0.7676 |
| **Variable** | **AUC** | **Specificity** | **Sensitivity** |
| **CDIST+SST+DEPTH** | **0.8769** | **0.8489** | **0.8195** |
| CDIST+SST+SLOPE | 0.8748 | 0.8528 | 0.8010 |
| CDIST+SST+ASPECT | 0.8726 | 0.8488 | 0.7920 |
| CDIST+SST+CHL | 0.8511 | 0.8286 | 0.7860 |
| CDIST+SST+CURL | 0.7532 | 0.7821 | 0.7150 |
| **Variable** | **AUC** | **Specificity** | **Sensitivity** |
| **CDIST+SST+DEPTH+SLOPE** | **0.8763** | **0.8467** | **0.8178** |
| CDIST+SST+DEPTH+ASPECT | 0.8744 | 0.8364 | 0.8175 |
| CDIST+SST+DEPTH+CHL | 0.8608 | 0.8268 | 0.8001 |
| CDIST+SST+DEPTH+CURL | 0.7498 | 0.6078 | 0.8681 |

Table S2. Covariate performance metrics used to inform variable selection for the winter of 2003 GAM model: Area Under the Curve (AUC), Specificity (correctly predicted absence locations); Sensitivity (correctly predicted presence locations). Covariate abbreviations as follows: Distance from colony (CDIST); chlorophyll concentration (CHL) and sea surface temperature (SST). Bold face indicates the covariates and their AUC, specificity and sensitivity values for the final model for each stage of cross-validation.

| **2003** | | | |
| --- | --- | --- | --- |
| **Variable** | **AUC** | **Specificity** | **Sensitivity** |
| **CDIST** | **0.7643** | **0.6330** | **0.8155** |
| DEPTH | 0.7311 | 0.7205 | 0.7245 |
| CHL | 0.7058 | 0.6711 | 0.7549 |
| CURL | 0.6815 | 0.7403 | 0.7086 |
| SST | 0.6272 | 0.3964 | 0.9050 |
| SLOPE | 0.5847 | 0.6445 | 0.5252 |
| ASPECT | 0.5508 | 0.6357 | 0.4897 |
| **Variable** | **AUC** | **Specificity** | **Sensitivity** |
| **CDIST+DEPTH** | **0.8008** | **0.7250** | **0.8063** |
| CDIST+CHL | 0.7780 | 0.6463 | 0.8462 |
| CDIST+SST | 0.7699 | 0.6324 | 0.8226 |
| CDIST+SLOPE | 0.7661 | 0.6406 | 0.7958 |
| CDIST+ASPECT | 0.7637 | 0.6233 | 0.7936 |
| CDIST+CURL | 0.6391 | 0.4459 | 0.9124 |
| **Variable** | **AUC** | **Specificity** | **Sensitivity** |
| **CDIST+DEPTH+CHL** | **0.8116** | **0.7259** | **0.8037** |
| CDIST+DEPTH+SST | 0.8073 | 0.6677 | 0.8517 |
| CDIST+DEPTH+ASPECT | 0.8020 | 0.7057 | 0.8089 |
| CDIST+DEPTH+SLOPE | 0.8007 | 0.7244 | 0.8055 |
| CDIST+DEPTH+CURL | 0.7135 | 0.6248 | 0.8146 |
| **Variable** | **AUC** | **Specificity** | **Sensitivity** |
| **CDIST+DEPTH+CHL+SST** | **0.8222** | **0.7188** | **0.8247** |
| CDIST+DEPTH+CHL+ASPECT | 0.8120 | 0.7216 | 0.7959 |
| CDIST+DEPTH+CHL+SLOPE | 0.8105 | 0.7239 | 0.8046 |
| CDIST+DEPTH+CHL+CURL | 0.6856 | 0.5633 | 0.8174 |
| **Variable** | **AUC** | **Specificity** | **Sensitivity** |
| **CDIST+DEPTH+CHL+SST+SLOPE** | **0.8220** | **0.7116** | **0.8313** |
| CDIST+DEPTH+CHL+SST+ASPECT | 0.8216 | 0.6955 | 0.8414 |
| CDIST+DEPTH+CHL+SST+CURL | 0.7386 | 0.6102 | 0.8168 |

Table S3. Covariate performance metrics used to inform variable selection for the winter combined all data GAM model: Area Under the Curve (AUC), Specificity (correctly predicted absence locations); Sensitivity (correctly predicted presence locations). Covariate abbreviations as follows: Distance from colony (CDIST); chlorophyll concentration (CHL) and sea surface temperature (SST). Bold face indicates the covariates and their AUC, specificity and sensitivity values for the final model for each stage of cross-validation.

| **All** | | | |
| --- | --- | --- | --- |
| **Variable** | **AUC** | **Specificity** | **Sensitivity** |
| **CDIST** | **0.7727** | **0.6308** | **0.8087** |
| DEPTH | 0.7484 | 0.7915 | 0.6541 |
| CHL | 0.7082 | 0.6950 | 0.7148 |
| CURL | 0.6809 | 0.6161 | 0.8077 |
| SST | 0.5861 | 0.4287 | 0.8345 |
| SLOPE | 0.5707 | 0.6853 | 0.4668 |
| ASPECT | 0.5534 | 0.7137 | 0.4084 |
| **Variable** | **AUC** | **Specificity** | **Sensitivity** |
| **CDIST+DEPTH** | **0.8005** | **0.6422** | **0.8349** |
| CDIST+CHL | 0.7798 | 0.6797 | 0.7771 |
| CDIST+SST | 0.7764 | 0.6780 | 0.7654 |
| CDIST+SLOPE | 0.7761 | 0.6887 | 0.7499 |
| CDIST+ASPECT | 0.7741 | 0.6807 | 0.7360 |
| CDIST+CURL | 0.6834 | 0.5589 | 0.8061 |
| **Variable** | **AUC** | **Specificity** | **Sensitivity** |
| **CDIST+DEPTH+SST** | **0.8049** | **0.6980** | **0.7795** |
| CDIST+DEPTH+ASPECT | 0.8010 | 0.7029 | 0.7665 |
| CDIST+DEPTH+SLOPE | 0.8005 | 0.6459 | 0.8310 |
| CDIST+DEPTH+CHL | 0.7974 | 0.6316 | 0.8481 |
| CDIST+DEPTH+CURL | 0.7136 | 0.6128 | 0.7893 |
| **Variable** | **AUC** | **Specificity** | **Sensitivity** |
| **CDIST+DEPTH+SST+ASPECT** | **0.8058** | **0.6857** | **0.7821** |
| CDIST+DEPTH+SST+SLOPE | 0.8048 | 0.6742 | 0.8033 |
| CDIST+DEPTH+SST+CHL | 0.8023 | 0.6715 | 0.8132 |
| CDIST+DEPTH+SST+CURL | 0.7281 | 0.5723 | 0.8249 |
| **Variable** | **AUC** | **Specificity** | **Sensitivity** |
| **CDIST+DEPTH+SST+ASPECT+SLOPE** | **0.8060** | **0.6766** | **0.7907** |
| CDIST+DEPTH+SST+ASPECT+CHL | 0.8023 | 0.6933 | 0.7788 |
| CDIST+DEPTH+SST+ASPECT+CURL | 0.7318 | 0.5710 | 0.8318 |
| **Variable** | **AUC** | **Specificity** | **Sensitivity** |
| **CDIST+DEPTH+SST+ASPECT+SLOPE+CHL** | **0.8025** | **0.6950** | **0.7775** |
| CDIST+DEPTH+SST+ASPECT+SLOPE+CURL | 0.7317 | 0.5747 | 0.8292 |

**References - supporting information**

Forcada, J., and I. J. Staniland. 2009. 'Antarctic Fur Seal Arctocephalus gazella.' in W. F. Perrin, B. Wursig and J. G. M. Thewissen (eds.), Encyclopedia of Marine Mammals (Academic Press (Elsevier)).
